# A 3D-engineered porous conduit for peripheral nerve repair

**DOI:** 10.1038/srep46038

**Published:** 2017-04-12

**Authors:** Jie Tao, Yu Hu, Shujuan Wang, Jiumeng Zhang, Xuan Liu, Zhiyuan Gou, Hao Cheng, Qianqi Liu, Qianqian Zhang, Shenglan You, Maling Gou

**Affiliations:** 1 State Key Laboratory of Biotherapy and Cancer Center, West China Hospital, Sichuan University, and Collaborative Innovation Center for Biotherapy, Chengdu, Sichuan province, China; 2 School of Materials Science and Engineering, Sichuan University, Chengdu, Sichuan province, China; 3 Department of Neurosurgery, West China Hospital, Sichuan University, Chengdu, Sichuan province, China; 4 Sinopharm A-THINK Pharmaceutical Co., Ltd., Jilin province, China

## Abstract

End-to-end neurorrhaphy is the most commonly used method for treating peripheral nerve injury. However, only 50% of patients can regain useful function after treating with neurorrhaphy. Here, we constructed a 3D-engineered porous conduit to promote the function recovery of the transected peripheral nerve after neurorrhaphy. The conduit that consisted of a gelatin cryogel was prepared by molding with 3D-printed moulds. Due to its porous structure and excellent mechanical properties, this conduit could be collapsed by the mechanical force and resumed its original shape after absorption of normal saline. This shape-memory property allowed a simply surgery process for installing the conduits. Moreover, the biodegradable conduit could prevent the infiltration of fibroblasts and reduce the risk of scar tissue, which could provide an advantageous environment for nerve regeneration. The efficiency of the conduits in assisting peripheral nerve regeneration after neurorrhaphy was evaluated in a rat sciatic nerve transected model. Results indicated that conduits significantly benefitted the recovery of the transected peripheral nerve after end-to-end neurorrhaphy on the static sciatic index (SSI), electrophysiological results and the re-innervation of the gastrocnemius muscle. This work demonstrates a biodegradable nerve conduit that has potentially clinical application in promoting the neurorrhaphy.

Peripheral nerve injury, which affects more than 200 000 persons in the United States of America each year^1^, is a common clinical problem all over the world^2^. And it lowers the life quality of patients and causes an enormous socioeconomic burden^3^,^4^. In clinic, end-to-end neurorrhaphy is the most popular method when the nerve defect is less than 5 mm^5^,^6^. Despite considerable advances in microsurgical techniques, the recovery of normal sensory and motor functions which are influenced by the location and time are usually unsatisfactory by using neurorrhaphy alone^7^. To our knowledge, only 50% of patients could regain useful function after treating with neurorrhaphy^8^.Therefore, additional procedures are being used to assist the functional recovery of peripheral nerve after end-to-end neurorrhaphy.

To improve the functional recovery after end-to-end neurorrhaphy, a favorable environment surrounding the injured site is necessary. Conduits were used to provide a hospitable environment for the regeneration of the peripheral nerve and supporting glial cells^9^,^10^. For peripheral nerve regeneration, the conduits could provide an adequate scaffold for cell adhesion and axonal regeneration, and could be semi-permeable for the metabolic exchange (such as oxygen and waste products)^11^. In addition, the wall of the conduit usually can slow down the diffusion of the growth or trophic factors secreted by the nerve stumps^12^. A wide range of natural and synthetic materials have been developed for nerve conduits, such as PLA^13^, Chitosan^14^, and Gelatin^15^. Materials which constructed a guidance cue should be biocompatible, flexible and soft so as to provoke minimal inflammatory response. Gelatin with good biocompatibility and degradability has been widely used in biomedical application such as tissue engineering, drug delivery and *in vitro* 3D cell culture^16^,^17^,^18^. Ju-Ying Chang et al demonstrated that the EDC/NHS-fixed gelatin conduit was successful in bridging a large gap in sciatic nerve of the rat^15^. Taking advantages of 3D printing technology, we previously 3D printed a cellularized conduit to repair the defects in rats. The cell-adhesive gelatin conduits with a designed structure could release neurotrophic factors for peripheral nerve regeneration^19^. However, there were few of conduits that wrap around the injured nerve to assist the axonal regeneration after end-to-end neurorrhaphy.

Based on this knowledge, designing a 3D engineered conduit that wrapped around the surgical site would be a promising protocol to promote the function and histology of the sciatic nerve after end-to-end neurorrhaphy. Although the prepared conduit did not mainly support a channel for the proliferation of schwann cells and the axonal regeneration, they could play a role in offering an unperturbed environment for nerve regeneration, such as preventing fibrous scar tissue invasion, and permeating nutrients and oxygen^20^. Wrapping the transected nerve with gelatin conduits might provide a development to facilitate the regeneration of peripheral nerve after end-to-end neurorrhaphy. However, to wrap a conduit, we usually need surgical exposure of the peripheral nerve that matches the length of the conduit or need additional sutures, which would add the complexity for surgeon and was not beneficial for the recovery of the injured nerve. In order to simplify the surgical procedure, we constructed a shape-memory conduit. The prepared conduit could regain its original geometry and could be used for wrapping the sciatic nerve after end-to-end neurorrhaphy in rats (Fig. 1).

Taking advantage of aforementioned studies, the aim of this study was to construct a 3D-engineered porous conduit, to further evaluate its usefulness for peripheral nerve repair after end-to-end neurorrhaphy. We studied the bulk mechanical behavior, morphology, structure of the conduits. Finally, functional assessment and histological evaluation were utilized to evaluate the effect of the conduit.

## Results

### Synthesis and characterization of gelatin cryogels

**Figure 2:**
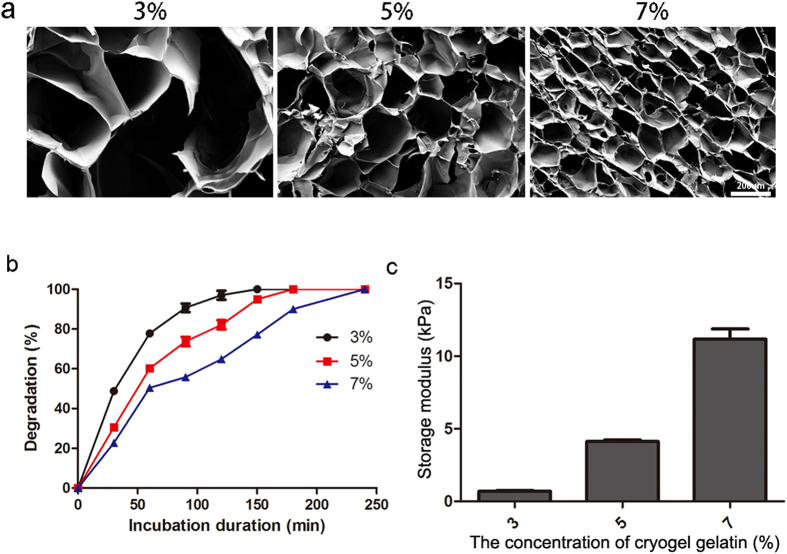
Characterization of gelatin cryogel. (**a**)SEM images of the cryogel with different concentration; (**b**) Degradation profiles upon incubation with collagenase; (**c**) Storage modulus for 3%, 5%, 7% (w/v) gelatin cryogel.

In order to select a proper concentration for preparing conduit, gelatin cryogels with different concentration (3%, 5%, 7%) were synthesized. One of the most important features of the cryogel was the microstructure which can affect many properties of the scaffolds. The microstructures of the gelatin cryogels were observed by SEM. The SEM images showed a uniformed porous structure throughout all the samples. The average size of pores decreased with the concentration from about 200 um and 150 um to 100 um in 3%, 5%, and 7% of the gelatin cryogels, respectively (Fig. 2a). The degradation profile of the gelatin cryogels was assessed in the presence of collagenase (100 ug/mL) by measuring the percentage of hydrogel residual mass at the indicated time point (Fig. 2b). This analysis revealed that gelatin cryogel could be degraded completely by collagenase solution in about 250 mins. Moreover, the degradation rate decreased with the concentration of gelatin. The mechanical properties of each prepared gelatin cryogel was tested by using an unconfined compression test (Fig. 2c). As expected, we detected that the storage modulus of the gelatin cryogels increased with its concentration. Collectively, the concentration of the gelatin was found to impact the physical and mechanical properties of the prepared gelatin cryogel. A higher concentration would result in stiffer and more durable hydrogels, with smaller pore size. The results suggested that some characterizations of material were controllable.

### Preparation and characterization of the porous conduit

**Figure 3:**
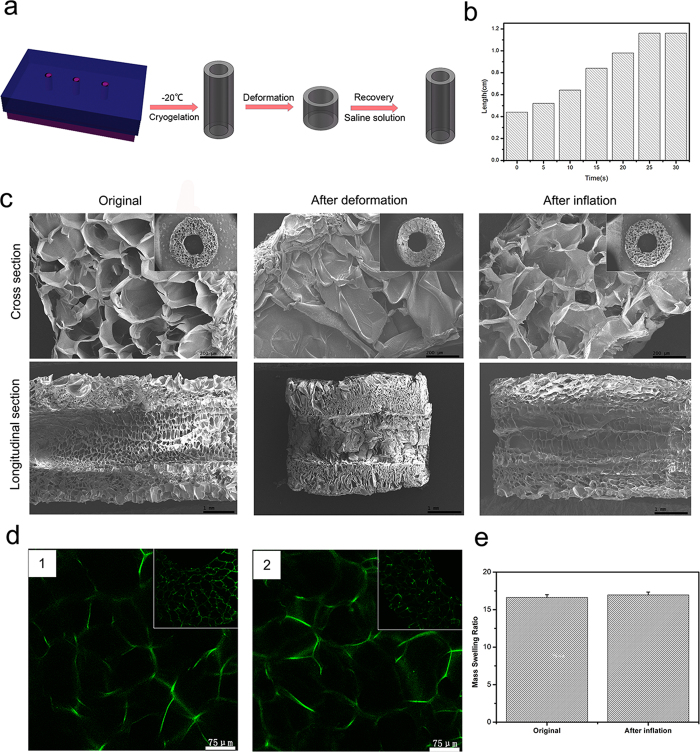
Characterization of the conduits. (**a**)Schematic illustration of the process of shape-memory property. (**b**) The change of the length of the shape-memory conduit with adding physiological saline. (**c**)The SEM images of different stages of the deformation. (**d**)The confocal laser scanning microscope image of the coumarin-6/MPEG-PLA nanoparticles in conduits. (**e**) Equilibrium swelling properties of the porous conduits at original and inflation state. Results are mean ± standard error of mean.

To prepare the conformal conduit, we fabricated some 3D printed moulds via 3D printing technology. The gelatin cryogel could be molded by the 3D printed moulds at −20 °C overnight. Due to the large pore size of the gelatin cryogel at a concentration of 3% and the difficulty of molding the gelatin cryogel at a concentration of 7%, the gelatin cryogel (5%) was used for preparing the conduits. The obtained conformal conduits had a length of 1 cm, an inner diameter of 1.5 mm and an external diameter of 4 mm, which could match the size of the sciatic nerve in rats. The mechanical force was added to compress its length and increase diameter, leading to a shape, like a ring. The fabricated conduits with a defined shape could regain their original geometry and architecture after added physiological saline (Fig. 3a). What’ more, the deformed conduit could regain its geometry in about 25 s *in vitro* (Fig. 3b). The shape-memory property of the conduit would promote the surgical process during the operation. The conduit with a simple preparation process, low cost and shape-memory property might be a potential scaffold for nerve regeneration.

To study the microstructures of the conduits from the original state to the recovery, we used scanning electron microscopy (SEM) to observe the cross and the longitudinal section of the conduit. The images shown in Fig. 3c presented that the conduit displayed a highly porous morphology and had pore size about 150 um at the original state. From the SEM images, we could detect that the morphology and the pore size of conduit did not change when the deformed conduit regained the shape. The deformed conduit was also observed by SEM, its surface was bestrewed with the fold. The pores of the conduit wall were collapsed and added together layer by layer. Since the processing used to repare the cryogels for SEM could cause expansion, confocal laser scanning microscope imaging of coumarin-nanoparticles was used to study the structure of these conduits in their hydrated state (Fig. 3d). The conduits demonstrated an interconnected pore structure and the structure of the conduit did not show significant difference after the conduit regained the shape, which was in accordance with the results of the SEM.

In addition, the freeze-dried conduits were incubated in PBS at room temperature over 24 h for reaching equilibrium, then the mass swelling ratio of the equilibrium mass to the dry mass was measured. As shown in Fig. 3e, the deformation almost had no effect on the mass swelling. The mass of the conduit in PBS was more than as triple as the mass of the freeze-dried.

**Figure 4:**
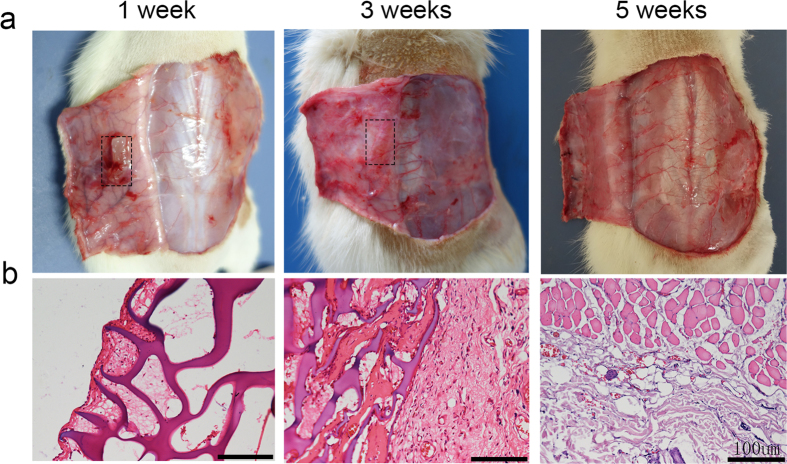
Degradation of conduits used for repairing the transected sciatic nerve in rat. The photographs and the representative H&E staining of tissue sections of the biodegradable conduits subcutaneously on the back of rats at 1, 3 and 5 weeks after implantation.

To assess the degradation of the shape-memory conduit *in vivo*, a rat implantation model was used. We took photos of the conduit, which was implanted subdermally on the back of rats at 1, 3, 5 weeks (Fig. 4a). The images at 1 week could be clearly seen that the conduit almost did not degrade. After 3 weeks, the conduit degraded partly. By week 5, we could not see the conduit in a visible form. The results indicated the degradation of the conduits *in vivo*. To evaluate the biocompatibility of the conduits, H&E staining was used (Fig. 4b). At 1 week, we could see rare fibroblasts and inflammatory cells in conduits, which suggested that the conduits suppressed the fibrous tissue ingrowth effectively and caused mild acute inflammatory responses. At 3rd week, the conduits elicited a foreign body reaction, and there was fibrous tissue that was observed in the conduits. At the 5th week, the conduit was completely degraded *in vivo*. Examination of the skin at the implanted site revealed normal subcutaneous tissue but no sign of the conduit. The results demonstrated that the conduit was degradable and biocompatible *in vivo*.

### The invasion of the fibroblasts and small molecule substance in gelatin cryogel

**Figure 5:**
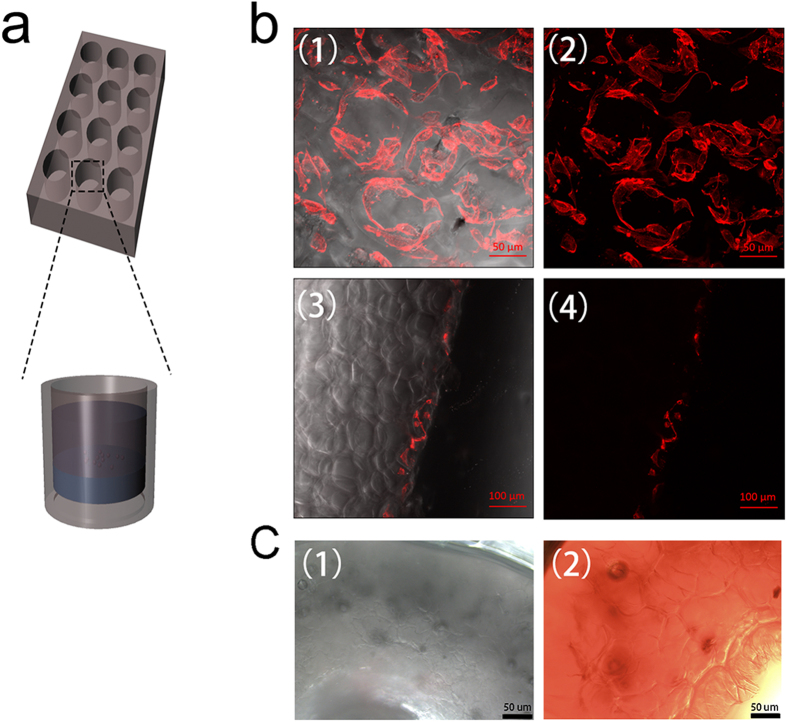
A model (**a**) designed for studying the invasion of NIH-3T3 cells in gelatin cryogel at a concentration of 5%. (**b**) the small molecule substance permeates in the conduit at 0 and 1 hour.

As an obstacle to traverse the injury site for axons, proliferation of scar tissue from the epineurium in response to injury will result in impediment to the regeneration. An ideal conduit had the ability to prevent the infiltration of the fibroblasts. To assess the invasion of NIH-3T3 cells in hydrated cryogel *in vitro*, the cryogel with 5% concentration was immersed into cell suspension and incubated for three days. Confocal microscope images showed that fibroblast cells could survive on the surface of the cryogel. The NIH-3T3 cells would attach around the wall of the pores. Longitudinal section of hydrogel revealed that the NIH-3T3 cells could not invade into the inner space during this period (Fig. 5a,b). The exchange of small molecule substance, such as oxygen and some waste products around the conduit might facilitate the regeneration of the peripheral nerve injures. We used vitamin B12 as a model to mimic the small molecule substance. In Fig. 5c, we found that the vitamin B12 could easily permeate in the conduit after 1 hour.

**Figure 6:**
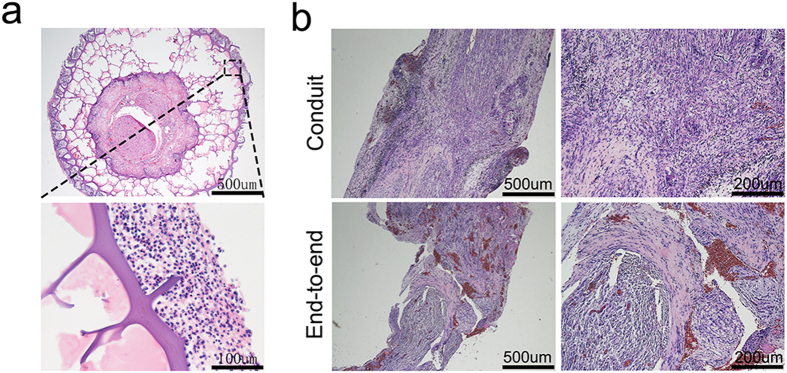
H&E images of sciatic nerve after (**a**) 3 days and (**b**) 20 days in experimental groups.

To assess the effect of the conduits *in situ*, the conduit was implanted in SD rats for wrapping the transected sciatic nerve. After 3 days, we took the nerve specimens from the experimental groups postoperative. H&E staining was performed on the transverse section of the nerve. As shown in Fig. 6a, the inflammatory cells stayed around conduit and we could not detect the fibroblasts or inflammatory cells in the middle of wall. This finding demonstrated that the porous structure of conduit could inhibit the infiltration of cells *in situ*. After 20 days, the nerve samples were harvested from the experimental groups for histological evaluation. The H&E staining for the longitudinal section of the regenerative nerve in Fig. 6b showed that the scar tissue in conduit group exhibited less in comparison with the end-to-end group. The results *in vitro* and *in vivo* indicated that the conduit could provide an advantageous environment for the regeneration of the injured peripheral nerve.

### Function recovery evaluation

**Figure 7:**
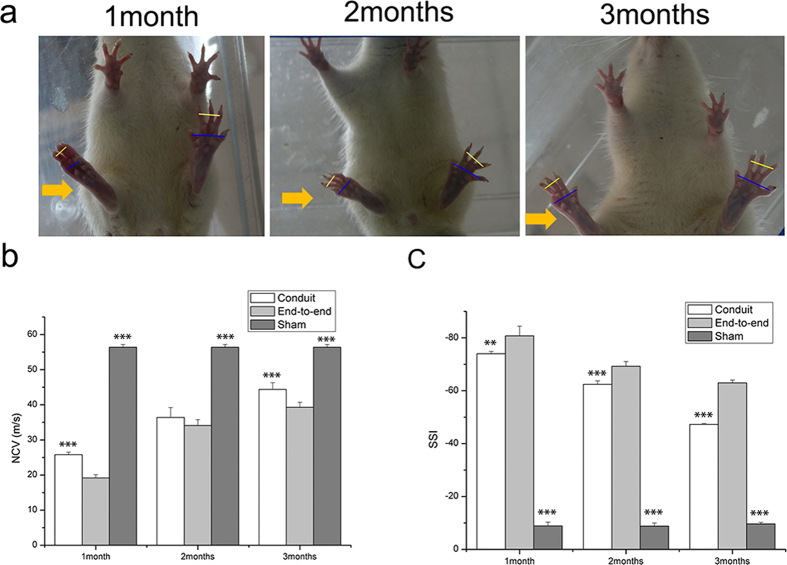
Functional evaluation of the regenerated nerve. (**a**) The view of the rats’ feet at 1,2,3 months. (**b**) Electrophysiological evaluation. Gastrocnemius electromyography in mice from various groups at 1,2,3 months after surgery. (c) Static sciatic index of different groups at 1,2.3 months after surgery. Results are mean ± standard error of mean. **P < 0.01, ***P < 0.005 vs end-to-end group.

In this study, conduits were fabricated for the regeneration of the sciatic nerve in rats. After sciatic nerve was repaired by end-to-end neurorrhaphy, the conduit rapidly regained its original shape and wrapped the injured site (Movie S1). After implanting the conduit around the transected nerve, we could observe beneficial effect on gait as the time went on (Fig. 7a). Static sciatic analysis and Electrophysiological test were used to assess the functional recovery of all operated animals and were quantified by calculating the static sciatic index (SSI) and nerve conduction velocity (NCV). SSI is a measure of the sciatic nerve function where a value close to 0 indicates normal function and a value close to −100 implies total impairment. Electrophysiology is a traditional method to assess the nerve regeneration after nerve injury and repair. Motor functional recovery was achieved in all groups at 1, 2, 3 months after surgery.

The electrophysiological analysis was assessed by calculating the nerve conduction velocity. The value of NCV at predefined time periods was shown in Fig. 7b. The NCV in sham group was about 56.4 m/s, which was significantly higher than in experimental groups. In experimental groups, the value of NCV in conduit group was higher than in the end-to-end group (39.3 ± 1.4 m/s)in the course of time. After 3 months, the NCV of the conduit group was 44.4 ± 1.9 m/s, close to the sham group (56.4 ± 0.8 m/s).

SSI check was performed and the results were shown in Fig. 7c. From the results of the walking tract analysis, the conduit group showed a higher SSI score than the end-to-end group. In the sham group, SSI value was −9.62 ± 0.55. After 3 months, animals in conduit group achieved a mean value for SSI of −47.26 ± 0.36, whereas in end-to-end group a mean value of −62.97 ± 1.1 was found. There was an insignificant change in the sham group. The improvement in SSI and NCV indicated that the application of conduit after peripheral nerve transection facilitated the functional recovery.

### Histological evaluation

**Figure 8:**
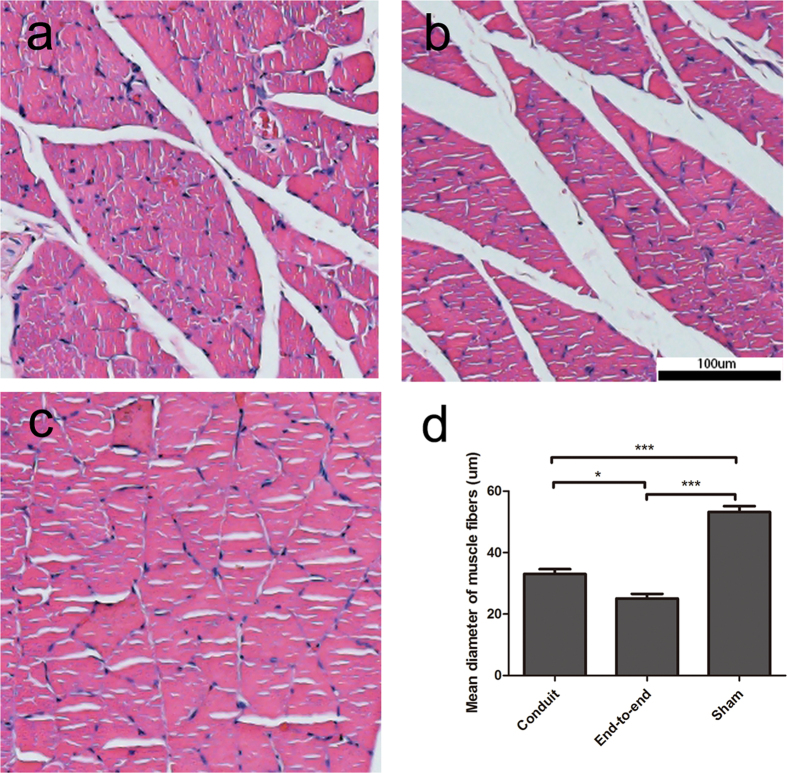
Histological analysis of target gastrocnemius muscles in each group. Representative light micrographs of the transverse-sectioned gastrocnemius muscle following H&E staining for the operated limb (**a**)in conduit group, (**b**)end-to-end group, (**c**)sham group at 3 months postoperatively. (**d**)The average percentage of muscle fiber in each group (n = 6, *p < 0.05, ***P < 0.005).

H&E staining was performed on the transverse section of the gastrocnemius muscles (Fig. 8). Due to peripheral nerve injury, muscle was denervated and the tendency of muscle degradation would occur. Compared to normal muscle morphology in sham group, gastrocnemius muscles which suffered from surgery were degenerated in experimental groups and showed a smaller muscle cell sectional area. The images of H&E sections showed that muscle fiber in sham group (58 ± 6 mm) had the largest diameter in all groups. The mean diameter of gastrocnemius muscle fibers in conduit group (33.08 ± 2.63 mm) were significantly larger than that in end-to-end group (25.08 ± 2.56 mm). The results indicated that the conduits were able to partially prevent muscle atrophy.

### Immunohistochemical evaluation

**Figure 9:**
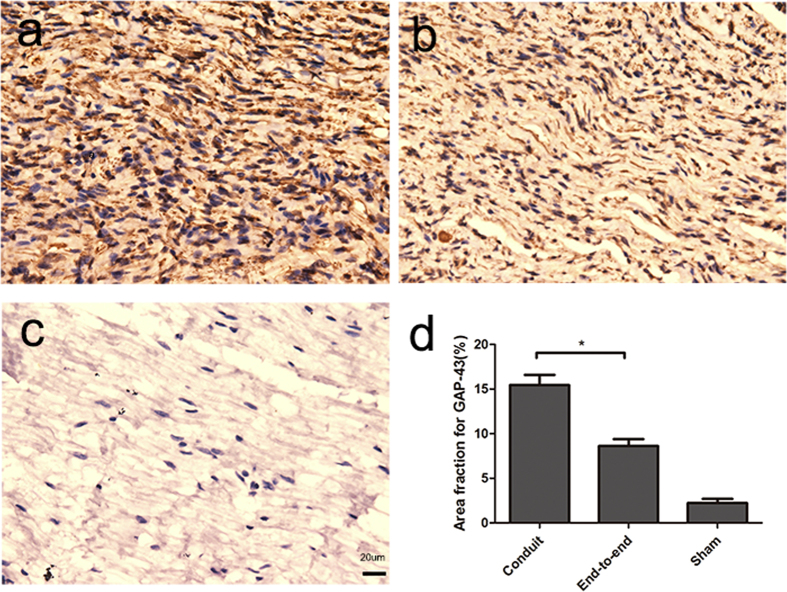
Histological analysis of GAP-43 expression as determined by immunohistochemistry on longitudinal tissue sections: (**a**)conduit group (**b**) end-to-end group (**c**) sham group (**d**) Graphic representation of the area fraction of results of the positive reaction of GAP-43 protein (n = 6, *P < 0.05).

Growth-associated protein (GAP-43) is widely described as the neuronal makers and an axonal growth maker during development. During regeneration and development of the peripheral nerve in the adult rats, GAP-43 is expressed at particularly high levels and assists neuronal pathfinding and branching. Immunohistochemistry was performed to evaluate the levels of GAP-43 on 2 months in all groups. As shown in Fig. 9, the immunohistochemical analysis of the proteins GAP-43 varied among the experimental groups and in comparison to the sham group. we observed a specific and localized positive reaction for GAP-43 within a few nerve fibres in the sham group, with a mean area fraction of 2.23%. In the experimental groups, we found positive expression of GAP-43 in the operative area. In the conduit group, we observed an intense and abundant positive reaction for GAP-43 associated with the newly formed nerve fascicles in the operation area. The area fraction of positive reaction in conduit group (15.44%) was larger than in end-to-end group (8.61%). All experimental groups showed a significant increase of the GAP-43 area fraction in comparison to the sham-operation group (p < 0.05). The results suggested that the 3D-engineered conduit could promote the regeneration of the injured sciatic nerve in rats.

## Discussion

The goal of using conduits is to promote the reconstruction of nerve continuity and functions^21^. The function recovery of peripheral nerve is closely associated with the biomaterials, which was chosen for conduit fabrication, and the microenvironment surrounding the injury site^22^. 3D printing technology with the ability to print the customized structure has been widely used in biomedical application^23^,^24^. In this study, by the 3D-printed moulds, a 3D-engineered conduit that consisted of gelatin cryogel was prepared. Owing to its porous structure, the obtained conduit could be deformed by the mechanical force and could rapidly regain its original shape with solution physiologique. The conduits were employed to wrap the transected sciatic nerve in rats. From 1 to 3 months, the regenerative capacity of these conduits was evaluated by a series of measurements. The recovery in the motor function of the injured sciatic nerve in conduit group, which was measured by the SSI value and NCV, was closer to that in sham group as time went on, and prevailed over in end-to-end group. Histological assessment showed that the target gastrocnemius muscle in conduit group achieved significantly better reconstruction than in end-to-end group, both in qualitative and quantitative aspects. Our finding suggested that conduits have the ability of enhancing the repair capacity of peripheral nerve. The prepared conduit with low cost, and readily operation may lead to the development of future 3D-engineered conduit for clinical use after neurorrhaphy.

For the interconnected microporous structure, highly elasticity and excellent mechanical property, cryogels have been introduced as promising biomaterials for biomedical application, such as cell culture, wound repair, and cancer vaccines delivery^25^,^26^,^27^. Cryogelation is the technique for fabrication the cryogel^28^. During the cryogelation, the growing ice crystals perform as porogen. After complete polymerization, the ice crystal will melt at room temperature, and the interconnected porous structure will be formed. The interconnected porous structure is one of the most important properties of the cryogels. The macroporosity in cryogels makes permeation of high molecular weight solutes and nutrients as well as transport of cellular waste^29^. For tissue engineering, many studies suggested that the interconnected porous structure could offer an excellent substrate for axon regeneration^30^. The conduit with a porous structure not only provided a room to permeate the oxygen and nutrients but hindered the fibroblasts’ invasion^31^. Ying Wan *et al* prepared a porous conduit which could maintain regularly tubal shape and retain the compressive strength after 10-week implantation in rabbits. This kind of conduit have promising potential for long gap nerve repair^32^. So, it is potential to apply a porous conduit to promote the regeneration of peripheral nerve. To facilitate neurorrhaphy for peripheral nerve repair, we fabricated the porous conduits using the gelatin cryogel. With controlled mechanical strength, pore size, and the degradation speed, the gelatin cryogel might be applied to different environment. The fabricated conduit showed a high biocompatibility and biodegradability *in vivo,* which was in accordance with previous study^33^.

For the specific property of the cryogels, they often regain their original shape after deformation^34^,^35^. Taking advantage of shape-memory property of the cryogels, researchers utilized shape-memory scaffolds for minimally invasive surgical approaches^36^,^37^. The scaffolds could be injected through a conventional needle while rapidly reassumed their original shape when injected to the targeted site. In our study, the prepared 3D-engineered porous conduit with original shape, which was implanted for wrapping the sciatic nerve in rats, would result in isolating more sciatic nerve. To promote the surgical process, we fabricated a shape-memory conduit using gelatin cryogel. Depending on the property of the cryogel, this conduit could resume the shape when an appropriate stress was applied. The large volumetric change of the conduit was caused by reversible collapse of the interconnected pores. The conduit could be compressed like a ring with mechanical force. Then water infusing into the hydrophilic gel released stored elastic energy and regained its original architecture in about 25 s. The water-responsive shape-memory behavior of conduits could simplify the surgical process and reduce the surgical time for surgeon. It provided an alternative protocol for conduit to wrap the injured peripheral nerve.

Previous studies demonstrated that the pore size of the conduits less than 30 um could be ideal for the regeneration of peripheral nerve. And the conduit wall could inhibit the invasion of fibroblasts and macrophagocytes^38^. Scar-forming fibroblasts might be the main cells that could damage the regeneration of peripheral nerve^39^. The fibroblasts can form the scar at the transected site, which inhibits the growth of the proximal axon^40^. In our study, to test the invasion of the fibroblasts, the NIH-3T3 cells were cultured on the surface of the gelatin cryogel. After 3 days, the results showed that the prepared gelatin cryogel could prevent the infiltration of cells, which was in accordance with the testing *in vivo*. Moreover, the 3D-engineered conduits prevented the scar *in vivo*. Although, the pore size of the cryogel (100 um in 5% gelatin cryogel) provided channels for the invasion of cells theoretically, the channels in the wall were flexural and far-flung. So, it would take much time for the cells to invade into inner site. In rats, the axonal elongation shows a rate of 1–3 mm/day^2^. Compared with bridging a long defect in rats, the small gap repairing by end-to-end suturing required less time. It might be one of the main reason why the conduit could inhibit the invasion of the fibroblasts and prevent the formation of scar.

During the development, GAP-43 played a key role in neurite outgrowth cone navigation. GAP-43 had been shown expressed in regenerating axons following nerve injury and played an important role in nerve sprouting post-operation^41^. The results of immunohistochemical evaluation showed that the positive area fraction for GAP-43 had a high expression in conduit group. The high expression of GAP-43 indicated that the conduit could promote the development and regeneration of the sciatic nerve in rats. When the growth factors were secreted by the stumps, the conduit with a highly porous structure could trap and slowly release them, which could favor the functional recovery.

## Conclusions

In summary, we constructed a 3D-engineered porous conduit to facilitate peripheral nerve regeneration. Because of the porous structure and controlled machinal property, the conduit owned the shape-memory property which allowed a readily surgical procedure for installing the conduit *in vivo*. This 3D-engineered porous conduits improved the regeneration of the injured nerve through providing a favorable environment and preventing the invasion of scar tissue. The 3D-engineered porous conduits used for wrapping the transected peripheral nerve demonstrated a promising strategy for the peripheral nerve regeneration after neurorrhaphy.

## Experimental Section

### Materials

Materials included Dulbecco’s modified Eagle’s medium (DMEM), Gelatin from porcine skin (Sigma, USA), EDC.HCl (1-Ethyl-3-(3-dimethylaminopropyl)carbodiimide hydrochloride) (Xiya Chemical Industry Co Ltd Shandong, China).

NIH-3T3 fibroblasts were cultured in DMEM with 10% (v/v) fetal calf serum, 100 U/ml penicillin, and 100 μg/ml streptomycin (Gibco) at 37 °C in a 5% CO_2_ atmosphere.

Sprague-Dawley (SD) rats were obtained from the Laboratory Animal Center of Sichuan University (Chengdu, China) and maintained under specific pathogen-free conditions. Rats were acclimatized to the environment of the animal facility for at least seven days prior to the experiments.

### Preparation of the gelatin cryogel

The gelatin cryogels were prepared as previously described^28^. Briefly, a calculated amount of gelatin was well dissolved in deionized water at 60 °C to form a gelatin solution (sol). Following, the gelatin solution was perfused into the 24- well plate with 1% of EDC·HCl (w/v). The solution was stored at −20 °C for 24 hours for cryopolymerization. During cryogel production under semi-frozen condition, the growing ice crystals performed as porogen. The obtained cryogels were washed with water and lyophilized.

### Preparation of the 3D-engineered porous conduit

The conduits were prepared using an indirect 3D printing technology as our previously described^19^. Briefly, 5% (w/v) of Gelatin was well dissolved in deionized water at 60 °C to form a gelatin solution (sol), following by injecting the solution with EDCI in the 3D-printed moulds. After cryogelation under semi-frozen condition, the conduit would be achieved. The hydrated gelatin conduits were washed by deionized H_2_O extensively. After immersed in 75% ethyl alcohol for one hour, the conduits were lyophilized overnight (Boyikang), and stored in –20 °C prior to use.

The length and the inner diameter of the conduits were deformed through the 3D printed moulds. Using the moulds, the nerve conduit could be deformed like a ring. The length of the conduit could be compressed to be one third of the original condition. The inner diameter could be expanded through the 3D -printed mould.

### SEM

To evaluate the microstructures of the gelatin cryogel with different concentrations and the conduit with different modes, the swollen hydrogel samples were frozen at −60 °C and following lyophilized. The dried samples coated with Pt/Pd were observed by using a scanning electron microscopy under an accelerating voltage of 5 Kv.

### Laser Confocal Microscopy

In order to characterize the hydrated conduits, we mixed coumarin-6 nanoparticles into gelatin solution for its specific fluorescence property. The preparation of coumarin-6 nanoparticles was described as our previous study^42^. The preparation method of nanoparticles-loaded conduits was the same as that of conduits. To evaluate the hydrated cryogel structure, coumarin-gelatin conduits were placed in dH2O on a glass slide, and imaged on a Leica SP5 laser scanning confocal microscope.

### Swelling ratio

To determine the effective swelling, the gelatin conduits were lyophilized and dry weights (Wi) were measured. Dried cryogel samples were immersed in 50 ml of diH2O and in incubated at 37 °C to reach equilibrium swelling state for 24 h. The samples were rinsed with diH2O and the swollen hydrogel weights (Ws) were measured. The swelling ratio (Q) was calculated by Q = Ws/Wi.

### Storage modulus

The storage modulus of swollen gelatin cryogels was measured using a dynamic mechanical analysis (DMA, Q800 TA Instruments). Samples of different concentration were incubated in diH2O at 37 °C for 48 h. the storage modulus of the gelatin cryogel was measured at an oscillation frequency of 1, 2, 5 Hz at 25 °C.

### Degradation

The degradation kinetics of the gelatin cryogel with different concentrations were determined from percent mass loss in response to enzymatic digestion. Gelatin cryogels were immersed in collagenase solution (100 ug/mL) in PBS on an orbital shaker at 37 °C, with the hydrated mass at increasing exposure time compared to its original mass.

To evaluate the degradation behavior *in vivo*, SD rats were employed. The shape-memory conduits were implanted subdermally on the back of SD rats. After implantation, the implanted sites were photographed to observe the shape-memory conduits at 1,3 and 5 weeks. The surrounding tissue were harvested together, fixed in 4% paraformaldehyde, embedded in paraffin, sectioned, and stained with hematoxylin and eosin (H&E) for further histopathological examination. We confirm that all experiments were performed in accordance with the guidelines and regulations of Sichuan University Committee on Animal Research and Ethics and were approved by the Institutional Animal Care and Use Committee of West China Hospital of Sichuan University.

### The invasion of NIH-3T3 cells and small molecule substance in gelatin cryogel

To characterize the invasion of NIH-3T3 cells in hydrated cryogels which had the same concentration with conduits, cells were harvested using a non- enzymatic cell dissociation solution (Sigma) and resuspended at 5*10^5^ cells/ml in complete medium. Cryogels were immersed in 500 μl of cell suspension in a 24 well-plate. After 3 days, samples were fixed in 4% paraformaldehyde solution for 24 h at room temperature. Then, the samples were washed with PBS three times following being immersed into 500 uL rhodamine phalloidin solution in the dark for 1 h. To evaluate the invasion of NIH-3T3 cells in hydrated cryogel, the samples with surface and longitudinal section were imaged using laser scanning confocal microscope.

To evaluate the effect of the gelatin cryogel for inhibiting the invasion of the fibroblasts and scar tissue *in vivo*, the conduits were implanted for wrapping the transected sciatic nerve in rats. The surgical procedure was the same as the following experimental group. After 3 and 20 days, the sciatic nerves with conduit were harvested for histological evaluation.

To assess the permeation of small molecule substance in conduit, vitamin B12 was set as a model substance. The conduit was immersed into a solution containing vitamin B12 (0.1 mg/mL). After 1 hour, we cut 1-mm part in middle of the conduit to take images under the light microscope.

### Animals and surgical procedure

Adult SD rats (200–250 g) were used to evaluate the nerve regeneration performance. The rats were divided into three groups: sham group (n = 9), conduit group (n = 18), end-to-end group (n = 18). The three group rats were anesthetized with intraperitoneal injections of chloral hydrate (0.5 ml/100 g). The right hindquarter was shaved; the skin was incised 1 cm posterior and parallel to the femur; and the biceps femoris was bluntly split to expose the sciatic nerve. The transection was performed 5 mm proximal to its trifurcation. The end-to-end neurorrhaphy was performed with 8–0 absorbable vicryl sutures and 1 suture. For conduit group, the porous conduits were used to wrap around the injured site, followed by end-to-end neurorrhaphy. Then the physiological saline was dropped on the conduits. For end-to-end group, the transected sciatic nerve was sutured by end-to-end neurorrhaphy. Following the implantation, the muscles were approximated, and the wound was closed by suturing the skin with 2–0 interrupted nylon sutures.

### Walking track analysis

To evaluate the behavior of the rats after peripheral nerve injury and repair, static sciatic analysis was performed at 1, 2, 3 months postoperatively. To take the images of the plantar surface of the rat’s paws, the rats were placed into the transparent container, which restricted their movements within the camera’s field of view. The images were analyzed using the software Image-Pro Plus. The toe spread (TS, distances between the first and fifth toe 1–5) and intermediate toe spread (ITS, distance between the second and fourth toe 2–4) were measured. According the previously described^21^, the SSI was calculated:


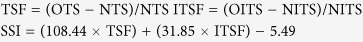


TSF: toe spread factor, OTS: operated side toe spread, NTS: non-operated side toe spread, ITSF: intermediate toe spread factor, OITS: operated side intermediate toe spread, NITS: non-operated side intermediate toe spread, SSI: static sciatic index.

### Electrophysiology

The electrophysiological analysis was conducted using previous method^43^. The electrophysiological evaluation was performed at 1 month intervals up to a period of 3 months using an electromyograph machine (Nuocheng, Shanghai, China) at room temperature. Before electrophysiological evaluation, all animals were deep general anesthesia with 10% chloral hydrate (0.5 ml/100 g) by intraperitoneal injection. We have evaluated the electrophysiology by surface stimulation, a bipolar stimulating electrode was placed between the proximal and distal ends of the regenerated nerves. Then a monopolar recording electrode was placed in the gastrocnemius muscle. After the above process, nerve conduction velocity across the regenerated nerve was recorded.

### Histological evaluation

For the histological analysis, the gastrocnemius muscle was harvested from each group at 3rd month post-implantation. The paraformaldehyde-fixed gastrocnemius muscles were dehydrated, embedded in paraffin. Following the embedding, histological slides were prepared using a transverse section in 5 um thickness throughout the half of the belly of each of the studied muscles. The slides were stained in a conventional manner, with hematoxylin and eosin, and subsequently images were taken of randomly selected high magnification fields from the light microscope (Leica DM4000B, W. Nuhsbaum, Inc., McHenry, IL, USA) for each muscle.

### Immunohistochemical evaluation

The regenerated nerve specimens were harvested at 2 months after surgery. The nerve paraffin sections of each group were also cut to 10 mm thickness for the immunohistochemistry analysis. The tissue was stained for immunohistochemistry to GAP-43. Slides were dewaxed in xylol, dehydrated in ethanol, and rinsed with PBS, followed by blocking with goat serum. Samples were incubated in a solution containing GAP-43 primary antibody. Samples were incubated overnight at 4 °C and visualized by DAB. After the process of dehydration, transparency and mounting, the nerve samples were observed under the upright microscope. To evaluate the positive ratios of GAP-43, the images were analyzed by the image analysis system.

### Statistical analysis

The data represent means with standard error of the mean and p < 0.05 were considered as statistically significant. Statistical tests were calculated using a One-Way ANOVA method, which was performed in a Graphpad Prism 5.00 software.

## Additional Information

**How to cite this article:** Tao, J. *et al*. A 3D-engineered porous conduit for peripheral nerve repair. *Sci. Rep.* **7**, 46038; doi: 10.1038/srep46038 (2017).

**Publisher's note:** Springer Nature remains neutral with regard to jurisdictional claims in published maps and institutional affiliations.

## Supplementary Material

Supplementary Movies S1

Supporting Information

## Figures and Tables

**Figure 1:**
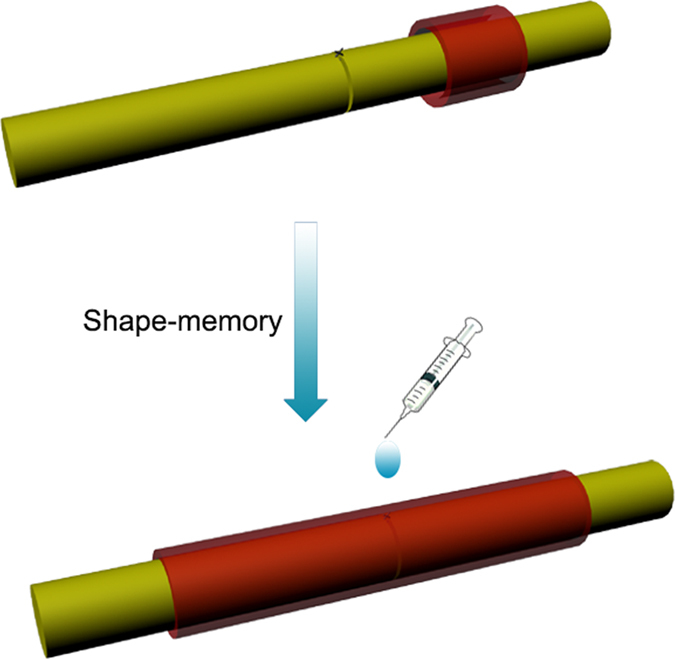
Schematic illustration of the process of the conduits for the transected peripheral nerve injury after end-to-end neurorrhaphy.
