# Supplementary material for: A 3D-engineered porous conduit for peripheral nerve repair
Source: Sci Rep. 2017 Apr 12;7:46038. doi: 10.1038/srep46038 (PMC5388843; doi:10.1038/srep46038)
Supplement: Supporting Information [file srep46038-s2.pdf]

A 3D-engineered porous conduit for peripheral nerve repair

Jie Tao[1,2,#], Yu Hu[3#], Shujuan Wang[1,4,#], Jiumeng Zhang[1],Xuan Liu[1], Zhiyuan Gou[1], Hao Cheng[1], Qianqi Liu[1], Qianqian Zhang[1], Shenglan You[1], Maling Gou[1*]

1 State Key Laboratory of Biotherapy and Cancer Center, West China Hospital, Sichuan University, and Collaborative Innovation Center for Biotherapy, Chengdu, Sichuan province, China

2 School of Materials Science and Engineering, Sichuan University, Chengdu, Sichuan 610065, China

3 Department of Neurosurgery, West China Hospital, Sichuan University, Chengdu, Sichuan province, China

4 Sinopharm A-THINK Pharmaceutical Co., Ltd

*Corresponding author: Maling Gou; email: goumaling@scu.edu.cn

#These authors contributed equally to this work and are co-first authors.

Movie. S1 The process of the 3D-engineered conduit for wrapping the sciatic nerve after end-to-end neurorrhaphy.
